# Integration of Metabolomics and Transcriptomics to Reveal the Metabolic Characteristics of Exercise-Improved Bone Mass

**DOI:** 10.3390/nu15071694

**Published:** 2023-03-30

**Authors:** Jin-Li Hou, Wan-Yu Yang, Qiong Zhang, Hao Feng, Xiao-Bao Wang, Hui Li, Sheng Zhou, Su-Mei Xiao

**Affiliations:** 1 Department of Epidemiology, School of Public Health, Sun Yat-sen University, Guangzhou 510080, China; 2 College of Marine Sciences, South China Agricultural University, Guangzhou 510642, China; 3 Guangdong Provincial Key Laboratory of Food, Nutrition and Health, School of Public Health, Sun Yat-sen University, Guangzhou 510080, China

**Keywords:** bone mass, swimming training, zebrafish, metabolomics, transcriptomics

## Abstract

(1) Background: Exercise is effective in promoting and maintaining bone mass. The aim of this study was to detect the exercise-induced metabolic changes in bone tissue of zebrafish. (2) Methods: Thirty-eight zebrafish (Danio rerio, six months old) were analyzed. The exercise group (*n* = 19) received 8 weeks of counter-current swimming training. The control group (*n* = 19) was not subjected to exercise. Mineralization was quantified, and alkaline phosphatase (Alp) and anti-tartrate acid phosphatase (Trap) activities were estimated (*n* = 12). The metabolomics (*n* = 12) and transcriptomics (*n* = 14) data of bone tissue were used for the integration analyses. (3) Results: The results showed that the exercise training improved the bone mineralization of zebrafish, e.g., the exercise group (5.74 × 10^4^ ± 7.63 × 10^3^) had a higher mean optical density than the control group (5.26 × 10^4^ ± 8.56 × 10^3^, *p* = 0.046) for the caudal vertebrae. The amount of mineralized matrix in scales of the exercised zebrafish was also higher (0.156 ± 0.012 vs. 0.102 ± 0.003, *p* = 0.005). Both histological staining and biochemical analysis revealed increased Alp activity (0.81 ± 0.26 vs. 0.76 ± 0.01, *p* = 0.002) and decreased Trap activity (1.34 ± 0.01 vs. 1.36 ± 0.01, *p* = 0.005) in the exercise group. A total of 103 different metabolites (DMs, VIP ≥ 1, fold change (FC) ≥ 1.20 or ≤0.83, *p* < 0.050) were identified. Alanine, aspartate and glutamate metabolism, β-alanine metabolism, pyrimidine metabolism, and pantothenate and CoA biosynthesis were the significantly enriched metabolic pathways (*p* < 0.050). A total of 35 genes (*q* ≤ 0.050 (BH), |Log2FC| ≥ 0.5) were coenriched with the 103 DMs in the four identified pathways. Protein–protein interaction network analysis of the 35 genes showed that *entpd3*, *entpd1*, and *cmpk2* were the core genes. (4) Conclusions: The results of this study suggest that alanine, aspartate and glutamate metabolism, β-alanine metabolism, pyrimidine metabolism, and pantothenate and CoA biosynthesis contributed to exercise-induced improvements in bone mass.

## 1. Introduction

Bone is a metabolically active tissue with multiple functions, namely providing structural support, protecting vital organs, facilitating movement, and regulating mineral metabolism [1]. Bone mass and strength reach peak values at around 30 years old, then decrease with age in middle-aged and elderly people [2]. Accumulating and maintaining sufficient bone mass is critical for reducing the risk of some bone metabolic diseases and achieving a high quality of life in the elderly [3].

Exercise and physical training have been proven to be effective in promoting and maintaining bone mass and strength [4,5]. In 2021, a systematic review and meta-analysis found that exercise increased bone mass at the lumbar spine and femoral neck in men >18 years old [6]. Another systematic review of randomized controlled trials showed that progressive resistance training improved femoral neck bone mass in 521 individuals at risk of fracture, with the bone mass being 0.02 g/cm^2^ higher in the intervention group than in the control group [7]. Ravnholt et al., reported that 7 weeks of intense intermittent running increased bone mass by 0.9% from the preintervention bone mass in previously untrained subjects [8]. Studies have also found that long-term lack of exercise or mechanical loading may cause bone loss and increase the risk of fracture [9,10].

The process by which exercise regulates bone metabolism is complex and far from being fully understood, especially the related metabolites. Studies using transcriptomic analysis have reported that exercise can regulate bone metabolism by modulating the expression of exercise-sensitive genes [11,12]. The metabolic and physiological responses to exercise have been increasingly researched in recent years. Metabolomic alterations result from a series of highly dynamic and interacting systems at the biomolecular level, namely genomic, transcriptomic, and proteomic systems. Metabolites, as the ultimate products, closely influence the phenotype. Studies have investigated the exercise-induced alterations in the metabolic profiles of various tissues, namely muscle [11,12], plasma [13,14,15], heart, and liver [11]. Some studies have also reported exercise-induced changes in the metabolites of patients with different disorders, namely neuromuscular fatigue [12], Alzheimer’s disease [13], and myocardial ischemia [16]. However, no study has investigated the influence of exercise on bone tissue metabolites. Nonetheless, several studies have investigated the changes in metabolic profiles related to bone disease or bone phenotype [17,18]. For example, in 2018, Zhao et al., identified that serum alanine, aspartate, and glutamate metabolism; taurine and hypotaurine metabolism; aminoacyl-tRNA metabolism; glutathione metabolism; and glycine, serine, and threonine acid metabolism were closely related to bone mass in 136 Caucasian women [18].

To study bone-related phenotypes and disease, animal models are generally used because of the difficulty in obtaining human bone tissue. Zebrafish has been widely used to study bone- and mineral-related diseases in recent years [19]. Several studies on the effect of exercise on bone mass were also conducted in zebrafish [20,21]. This species shares many features with mammals in terms of skeletal elements, osteogenic mechanisms, and bone matrix components [22]. Approximately 82% of the genes in the zebrafish genome are homologous to human disease-related genes, including genes involved in regulating the skeletal system [23]. The physiology, development, and metabolism of zebrafish are highly similar to those of mammals [24].

To better understand the exercise-induced metabolic changes in bone, in this study, we designed an 8-week counter-current swimming training regimen for zebrafish and investigated the training-related changes in the phenotype, metabolomics, and transcriptomics of bone tissue in the exercise group in comparison with the control group. Our assessment of changes in metabolites may provide new insights into the molecular mechanism of exercise-induced improvements in bone mass and bone health.

## 2. Materials and Methods

### 2.1. Zebrafish Husbandry

All procedures were performed with the approval of the University of Sun Yat-sen Animal Ethics Committee (Approval No. 2022-003). Adult male zebrafish (Danio rerio; *n* = 38; 6 months old) obtained from the Laboratory Animal Center, Sun Yat-sen University were randomly distributed among two plastic tanks (60  × 45  ×  25 cm; 19 fish per tank) containing dechlorinated water at 26 °C and under a 14 h light/10-h dark cycle. All of the adult zebrafish were fed commercial food (brine shrimp containing protein, amino acids, fats, and unsaturated fatty acids) to satiety twice a day. Satiation is defined as the point within a 5 min period at which fish are no longer actively searching for food [25,26].

### 2.2. Zebrafish Exercise Training

Two weeks later, each tank of fish was randomly designated as the exercise (*n* = 19) or control (*n* = 19) group. To ensure that the zebrafish in the exercise group went through exercise training, a customized two-channel swim tunnel (45 × 20 × 25 cm) was created, with each channel connected to an aquatic powerhead (Figure S1). The exercise group fish were then transferred from the home tank to the two-channel swim tunnel. Under the action of the aquatic powerhead, circulating water flow formed in the swim tunnel, and the exercise group zebrafish swam against the flow, simulating resistance movements. The swimming exercise regimen comprised 3 h of exercise per day for a period of 8 weeks. The resistance movements had two phases. The phase 1 acclimatization training was performed in the first week. During days 1–3, the speed was 0.2 m/s and increased to 0.3 m/s for days 4–6 and 0.4 m/s on day 7. The phase 2 swimming training was performed for 7 weeks, with a flow rate of 0.4 m/s. When the exercise training began (a steady flow of water was formed in the exercise channel), the tail beat frequency and amplitude increased considerably, which indicated that the zebrafish in the exercise group began to swim against the current actively [27]. For the control group, the zebrafish were not subjected to exercise. The control group zebrafish were also taken out from the home tank and put into a space with a similar volume to that of the two-channel swim tunnel without the water flow at the same time when the exercise group zebrafish were transferred. Both of the groups were fed, and their tank water was changed, at the same time.

### 2.3. Staining of Bone Tissue and Quantification of Bone Mineralization

The adult zebrafish were euthanized by placing them in MS-222 anesthetic agent (0.05%), then fixed with 4% paraformaldehyde for 12 h. Subsequently, scales and skin were carefully removed uniformly from the anterior area of either side of the fish body using Dumont^®^ stainless steel forceps (Dumont, La Sagne, Switzerland). For alizarin red S(ARS) staining of bone tissue [28], the zebrafish that had been stripped of their scales and skin were dehydrated in 50% ethanol–phosphate-buffered saline (PBS) for 10 min, then stained with 0.1% ARS in 0.5% KOH overnight. After washes in ultrapure water, the zebrafish were bleached in 1.5% H_2_O_2_–1.0% KOH for 12 h. Next, destaining was performed with different proportions of 0.5% KOH–glycerol (3:1, 1:1, 1:3), and the destained fish samples were stored in 100% glycerol at 4 °C.

The method for the quantitation of bone mineralization was the same as previously described [29]. Adult zebrafish were placed in a Petri dish (60 mm) containing 100% glycerol and visualized and photographed using an M205 FA stereo microscope (Leica, Wetzlar, Germany) equipped with a DFC310 FX camera (Leica, Wetzlar, Germany). All images were taken under identical conditions. Image-Pro Plus image analysis software version 6.0 (IPP 6.0, Media Cybernetics, Bethesda, MD, USA) was used to quantify the areas and integral optical density (IOD) of ARS staining. Bone mineralization was evaluated using the mean optical density (IOD sum/Area sum). Six zebrafish from each group were stained with ARS for bone density assessment. Scales from zebrafish were used for the quantification of mineralization and the detection of alkaline phosphatase (Alp) and anti-tartrate acid phosphatase (Trap) activities.

### 2.4. Quantification of Mineralization in Scales

As previously described [30], the explanted scales that had been fixed with 4% paraformaldehyde were washed with phosphate buffer solution and stained for 1 h at room temperature with ARS dissolved in a 1.0% KOH solution to a final concentration of 10 μg/mL. After aspiration of the unincorporated dye, the scales were washed with PBS and incubated overnight with 20 mM TRIS buffer containing 0.25 M EDTA (pH 8.0) as the extraction agent. The colored supernatant was transferred into 96-well plates, and the calcium content of the supernatant, reflecting that of the scales, was evaluated by measuring the absorbance at 450 nm with a microplate reader (GENios Plus, Tecan, Männedorf, Switzerland). Six zebrafish from each group were used for the quantification of mineralization.

### 2.5. Biochemical Analyses of Alp and Trap Activities in Scales

To measure Alp activity, after being fixed with 4% paraformaldehyde and washed with PBS, the explanted scales were incubated in 100 μL of an alkaline buffer (100 mM Tris–HCl, pH 9.5; 1 mM MgCl_2_; 0.1 mM ZnCl_2_) for 1 h with constant shaking. The reaction was then stopped by adding 50 μL of a 3 N NaOH–20 mM EDTA solution. Next, 150 μL of the colored solution was transferred to a new plate, and its absorbance was measured at 405 nm with a microplate reader (TECAN GENios Plus). The absorbance was converted into the amount of produced para-nitrophenol (PNP) using a standard curve [31].

To measure Trap activity, after being fixed with 4.0% paraformaldehyde solution and washed with phosphate buffer solution, the explanted scales were incubated in 100 μL of 20 mM para-nitrophenyl phosphate and 20 mM tartrate in 0.1 M sodium acetate buffer (pH 5.3) for 1 h with constant shaking. Trap activity was then measured in the same manner as Alp activity [31]. Six fish from each group were used for the biochemical analyses of Alp and Trap activities.

### 2.6. Histological Staining of Alp and Trap in Scales

For the histological staining of Alp and Trap, after being fixed with 4% paraformaldehyde solution and washed with PBS, the explanted scales were stained using a TRAP/ALP stain kit from Wako according to the manufacturer’s protocol [32]. Six fish from each group were used for this purpose.

### 2.7. Statistical Analysis of Bone Phenotypes

The mean optical density, mineralized matrix content, and Alp and Trap activity levels are all expressed as the mean and standard deviation. Data normality was determined using the Shapiro–Wilk test. Student’s *t* test was used to compare the differences between the exercise group and the control group. To reduce the batch differences of mean optical density due to different photographing times, a mixed linear model containing both fixed effects (e.g., intervention method) and random effects (e.g., photographing time) was used to evaluate the impact of exercise on bone mass. *p*-values less than 0.05 were considered statistically significant in this study.

### 2.8. Metabolomics

#### 2.8.1. Metabolite Extraction from Bone Tissue

Briefly, 60 mg of bone tissue (from the ribs and vertebral column) were weighed and subjected to metabolite extraction by directly adding 800 µL of precooled extraction reagent (methanol:acetonitrile:water (2:2:1, *v*/*v*/*v*)), followed by a mixture of internal standards for quality control (QC) of the prepared samples. After homogenizing the bone tissue mixture for 5 min using TissueLyser (JXFSTPRP, JingXin Technology, Shanghai, China), the samples were sonicated for 10 min and incubated at −20 °C for 1 h. The samples were then centrifuged for 15 min at 25,000 rpm at 4 °C, and the supernatants were collected for vacuum freeze drying. The metabolites were resuspended in 200 µL of 10% methanol and sonicated for 10 min at 4 °C, followed by centrifugation for 15 min at 25,000 rpm. Finally, the supernatants were transferred to autosampler vials for liquid chromatography–mass spectrometry (LC-MS) analysis. A QC sample was prepared by pooling identical volumes of each sample to evaluate the reproducibility of the whole LC-MS analysis process.

#### 2.8.2. Bone Tissue Metabolic Profiling Analysis by Ultra-High-Performance Liquid Chromatography-Tandem Mass Spectrometry (UPLC-MS/MS)

In this analysis, a Waters 2D UPLC (Waters, Milford, MA, USA) tandem Q-Exactive high-resolution mass spectrometer (Thermo Fisher Scientific, Waltham, MA, USA) was used in simultaneous positive electron spray ionization (ESI+) and negative electron spray ionization (ESI−) mode for the separation and detection of bone tissue metabolites. Chromatographic separation was performed on a Waters ACQUITY UPLC BEH C18 column (1.7 μm, 2.1 mm × 100 mm; Waters), and the column temperature was maintained at 45 °C. In the positive mode, the mobile phase consisted of 0.1% formic acid (A) and acetonitrile (B), while in the negative mode, the mobile phase consisted of 10 mM ammonium formate (A) and acetonitrile (B). The gradient conditions were as follows: 0.0–1.0 min, 2% B; 1.0–9.0 min, 2–98% B; 9.0–12.0 min, 98% B; 12.0–12.1 min, 98–2% B; and 12.1–15.0 min, 2% B. The flow rate was 0.35 mL/min, and the injection volume was 5 μL. The MS settings for the positive and negative ionization modes were as follows: the spray voltages in the positive and negative ionization modes were 3.80 and 3.20, respectively; the sheath gas flow rate was 40 arbitrary units (arb); the aux gas flow rate was 10 arb; the heater temperature of aux gas was 350 °C; and the capillary temperature was 320 °C. The full scan range was 70–1050 *m*/*z* with a resolution of 70,000. To obtain more reliable experimental results during instrument testing, the samples were randomly ordered to reduce system errors. A QC sample was interspersed every 10 samples.

#### 2.8.3. Bone Tissue Metabolomic Data Processing and Annotation

The LC-MS/MS raw data (raw file) were imported into Compound Discoverer 3.1 (Thermo Fisher Scientific, USA) for data processing, and data on the compound molecular weight, retention time, peak area, and identification results were exported. To obtain high-quality ions, the relative standard deviation (RSD) of each ion peak was calculated, and ion peaks with RSD values higher than 30% were excluded. The identification of metabolites was based on a combined result from the BMDB (Bovine Metabolome Database) and the mzCloud and ChemSpider (HMDB, KEGG, and LipidMaps) databases. Partial least squares method discriminant analysis (PLS-DA) [33,34] was used to establish a relationship model between metabolite expression and sample groups. To evaluate the PLS-DA model, it was subjected to 200 response permutation tests. The VIP (variable importance in projection) values of the first two principal components of the PLS-DA model, together with the fold change (FC) and the *p*-value of Student’s *t* test, were used to select the differential metabolites (DMs) between the two groups. To include as many DMs as possible, they were selected when the following criteria were met: VIP values of the first two principal components of the PLS-DA model ≥ 1, FC ≥ 1.20 or ≤0.83, and *p*-values < 0.05. Metabolic pathway enrichment analysis of the DMs was performed using the Kyoto Encyclopedia of Genes and Genomes (KEGG) database. Metabolic pathways with *p*-values < 0.05 were considered to be significantly enriched by the DMs. Six fish from each group were used as individual samples for the metabolomic analysis. Table S1 shows the distribution for each zebrafish in the metabolomic analyses.

### 2.9. Transcriptomics

#### 2.9.1. Sample Preparation and RNA Extraction

Total RNA was extracted from 60 mg of zebrafish bone (from the ribs and vertebral column) using TRIzol reagent (Invitrogen, Carlsbad, CA, USA) following the manufacturer’s instructions. The OD260/230 ratio and the RNA integrity number were used to evaluate the quantity and quality of total RNA using a NanoDrop and an Agilent 2100 Bioanalyzer (Thermo Fisher Scientific, MA, USA).

#### 2.9.2. Library Construction and RNA Sequencing

Qualified total RNA was purified by DNase I, and mRNA was enriched by adding Oligo(dT)-attached magnetic beads. Subsequently, fragment buffer was added to cleave the mRNA into small fragments. Fragmented mRNA was used as a template for reverse transcription to generate the first cDNA strand; then, the second cDNA strand was generated. End repair and adaptor ligation were performed by adding A-tailing mix and RNA index adapters; then, PCR amplification was performed. A single-strand circle DNA library was obtained by recovering and cycling the PCR products. The final library was obtained by purifying the linear DNA molecules that were not cycled. The quality of the final library was tested. The qualified library was replicated by rolling circle replication to form DNA nanospheres (DNBs). DNBs were added to the mesh holes of a nanochip using high-density DNA nanochip technology. Subsequently, sequencing was performed on the BGIISEQ500 platform (BGI-Shenzhen, Guangdong, China) using combined probe-anchored polymerization.

#### 2.9.3. Read Filtering and Mapping

To obtain clean reads, the sequencing data were filtered using SOAPnuke (v1.5.2) [35] by removing (1) reads containing sequencing adapter, (2) reads whose low-quality base ratio (base quality less than or equal to 5) was more than 20%, and (3) reads whose unknown base (‘N’ base) ratio was more than 5%. Thereafter, the high-quality clean reads were stored in FASTQ format. The clean reads were mapped to the D. rerio genome (GCF_000002035.6_GRCz11) using HISAT2 (v2.0.4) [36]. Later, Bowtie2 (v2.2.5) [37] was applied to align the clean reads to the gene set from the genomic database for D. rerio built by the Beijing Genomics Institute, coding transcripts were included, and the gene expression levels were calculated using RNA-seq by Expectation Maximization (v1.2.12) [38].

#### 2.9.4. Analysis of Differentially Expressed Genes (DEGs)

DEGs were identified by DESeq2 (V1.4.5) as having a *p*-adjusted < 0.05 and |Log2 FC| > 0.5. Benjamini–Hochberg (BH) methods were chosen as the multiple test correction method to correct the obtained *p*-values. Seven fish from the exercise group and six fish from the control group were used as individual samples for the transcriptomic analysis. Table S1 shows the distribution for each zebrafish in the transcriptomic analyses.

#### 2.9.5. Validation of Transcriptome Analysis Using Quantitative Real-Time PCR (qRT-PCR)

RNA samples were prepared in the same manner as RNA-seq samples, as described above. qRT-PCR was performed using a QuantScript RT kit (Tiangen Biotech Co., Ltd., Beijing, China). *β-actin* was used as an internal reference gene. Detailed information on the primer sequences of *entpd3*, *entpd1*, *cmpk2*, and *β-actin* is shown in Table S2. Three replications were conducted for each group.

## 3. Results

### 3.1. Exercise Increased Zebrafish Bone Mass

**Figure 1:**
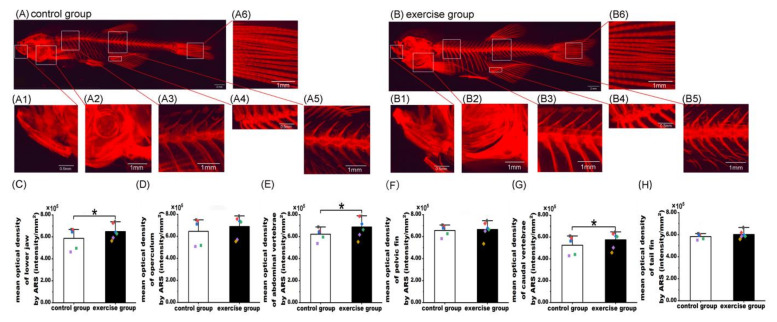
Alizarin red S (ARS) staining of bone tissue and quantification of bone mineralization in the control and exercise groups. Fluorescent image of ARS staining of zebrafish to visualize the whole-body skeleton of the control (**A**) and exercise (**B**) groups. Magnified fluorescent images of the lower jaw (**A1**,**B1**), operculum (**A2**,**B2**), abdominal vertebrae (**A3**,**B3**), pelvic fin (**A4**,**B4**), caudal vertebrae (**A5**,**B5**), and tail fin (**A6**,**B6**). Comparison of mean optical density in the lower jaw (**C**), operculum (**D**), abdominal vertebrae (**E**), pelvic fin (**F**), caudal vertebrae (**G**), and tail fin (**H**) between the exercise and control groups. The values of mean optical density are expressed as mean and standard deviation. A mixed linear model containing both fixed effects (e.g., intervention method) and random effects (e.g., photographing time) was used to evaluate the impact of exercise on bone mineralization in the lower jaw, operculum, abdominal vertebrae, pelvic fin, caudal vertebrae, and tail fin. The black, red, blue, green, and purple squares represent the control group fish numbers 13, 14, 15, 16, and 17, respectively. The black, red, blue, green, purple, and yellow diamonds represent exercise group fish numbers 14, 15, 16, 17, 18, and 19, respectively. *, *p* < 0.05.

After 8 weeks of swimming training, the bone tissues of zebrafish from the exercise and control groups were stained with ARS (Figure 1). Visually, the staining signal of the whole body in the exercise group (Figure 1B) was moderately stronger than that in the control group (Figure 1A). Furthermore, under high magnification, compared with the control group, the staining signals of the lower jaw (A1 and B1), operculum (A2 and B2), abdominal vertebrae (A3 and B3), caudal vertebrae (A4 and B4), pelvic fin (A5 and B5), and tail fin (A6 and B6) were increased in the exercise group (Figure 1).

To quantify the skeletal mineralization of zebrafish in both groups, IPP 6.0 was used to measure the area and IOD in the same areas of the lower jaw (A1, B1), operculum (A2, B2), abdominal vertebrae (A3, B3), caudal vertebrae (A4, B4), pelvic fin (A5, B5), and tail fin (A6, B6) in zebrafish (Figure 1). The mean optical density (IOD intensity/mm^2^) was used to evaluate the bone mineralization of the lower jaw, operculum, abdominal vertebrae, pelvic fin, caudal vertebrae, and tail fin in zebrafish of the two groups. As shown in Figure 1, eight weeks of exercise training significantly increased the mean optical density in the lower jaw (Figure 1C; control group, 5.87 × 10^4^ ± 9.92 × 10^3^, *n* = 5; exercise group, 6.48 × 10^4^ ± 7.06 × 10^3^, *n* = 6, *p* = 0.014), abdominal vertebrae (Figure 1E; control group, 6.22 × 10^4^ ± 5.79 × 10^3^, *n* = 5; exercise group, 6.87 × 10^4^ ± 9.44 × 10^3^, *n* = 6, *p* = 0.018), and caudal vertebrae (Figure 1G; control group, 5.26 × 10^4^ ± 8.56 × 10^3^, *n* = 5; exercise group, 5.74 × 10^4^ ± 7.63 × 10^3^, *n* = 6, *p* = 0.046). The differences of mean optical density in the other skeletal sites were not significant (*p* > 0.050), while their mean values in the exercise group were higher than those in the control group, e.g., in the operculum (Figure 1D; control group, 6.45 × 10^4^ ± 1.22 × 10^4^, *n* = 5; exercise group, 6.89 × 10^4^ ± 9.99 × 10^3^, *n* = 6), pelvic fin (Figure 1F; control group, 6.56 × 10^4^ ± 5.14 × 10^3^, *n* = 5; exercise group, 6.65 × 10^4^ ± 7.29 × 10^3^, *n* = 6), and tail fin (Figure 1H; control group, 5.83 × 10^4^ ± 2.54 × 10^3^, *n* = 5; exercise group, 6.02 × 10^4^ ± 3.66 × 10^3^, *n* = 6).

### 3.2. Exercise Increased Mineralization in Zebrafish Scales

**Figure 2:**
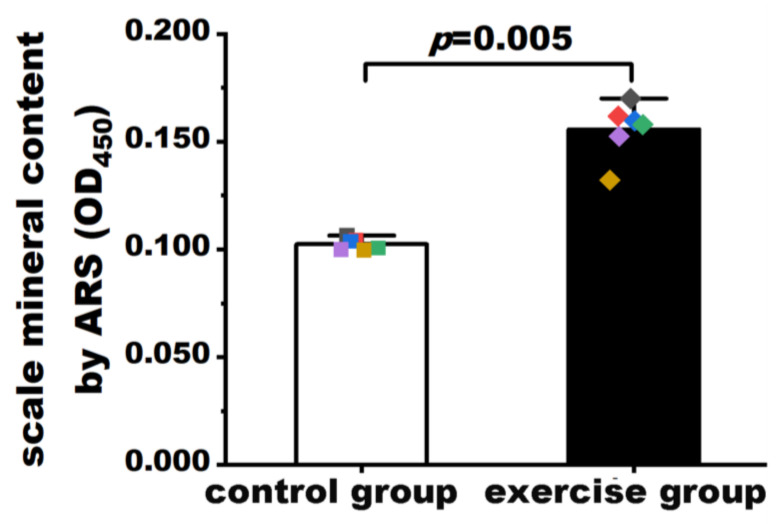
Quantification of mineralization in scales of the exercise and control groups. Values are expressed as mean and standard deviation. Student’s *t* test was used to compare the differences in mineralization levels between the exercise group and the control group. The black, red, blue, green, purple, and yellow squares represent control group fish numbers 13, 14, 15, 16, 17, and 18, respectively. The black, red, blue, green, purple, and yellow diamonds represent exercise group fish numbers 14, 15, 16, 17, 18, and 19, respectively.

The results of the quantification of mineralization in zebrafish scales are shown in Figure 2. The explanted scales of six fish from each group were incubated with ARS. The dye was then extracted by treatment with EDTA and read at 450 nm with a microplate reader. As shown in Figure 2, the mineralization levels in zebrafish scales were significantly higher in the exercise group (0.156 ± 0.012, *n* = 6) than in the control group (0.102 ± 0.003, *n* = 6, *p* = 0.005). The result of the permutation test (*p* = 0.001) was consistent with that of the Student’s *t* test.

### 3.3. Exercise Induced Alp Activity and Inhibited Trap Activity in Zebrafish

**Figure 3:**
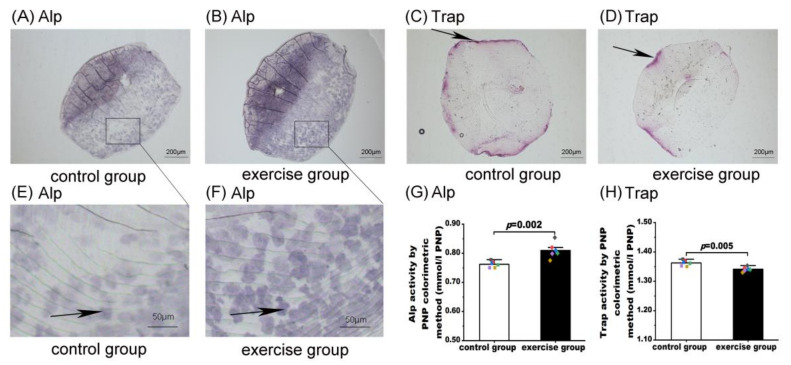
Measurements of alkaline phosphatase (Alp) and anti-tartrate acid phosphatase (Trap) activities in the control and exercise groups. Results of histological staining of Alp activities (**A**,**B**) and Trap activities (**C**,**D**; black arrows indicate the resorption activity along the anterior/lateral edge); closer views of histological staining of Alp activities (**E**,**F**; black arrows indicate the ALP-positive cells); results of biochemical assay of Alp activities and Trap activities (**G**,**H**). Values are expressed as mean and standard deviation. Student’s *t* test was used to compare the differences in biochemical activities of Alp and Trap between the exercise group and the control group. The black, red, blue, green, purple, and yellow squares represent control group fish number 13, 14, 15, 16, 17, and 18, respectively. The black, red, blue, green, purple, and yellow diamonds represent exercise group fish numbers 14, 15, 16, 17, 18, and 19, respectively.

The results of Alp and Trap activity assessments are shown in Figure 3. The explanted scales of six fish from each group were used for histological and biochemical analyses of Alp and Trap activities. Alp is involved in osteoblastic differentiation, while Trap is an osteoclast-specific marker enzyme [39,40]. As shown in Figure 3, compared with the control group (Figure 3A,E), the matrix-depositing cells showed stronger positive staining for Alp activity in the exercise group (Figure 3B,F). Biochemical analysis also showed that Alp activity in the exercise group (0.81 ± 0.26, *n* = 6) was significantly higher than that in the control group (0.76 ± 0.01, *n* = 6, *p* = 0.002, Figure 3G). For Trap activity, the Trap-positive signal along the borders of zebrafish scales in the control group (Figure 3C) was stronger than that in the exercise group (Figure 3D). Biochemical analysis also showed that Trap activity in the control group (1.36 ± 0.01, *n* = 6) was significantly higher than that in the exercise group (1.34 ± 0.01, *n* = 6, *p* = 0.005, Figure 3H). The results of the permutation test of the biochemical analysis of Alp activity (*p* = 0.003) and Trap activity (*p* = 0.005) were also consistent with those of the Student’s *t* test.

### 3.4. Metabolic Profiling of the Two Groups

LC-MS/MS technology was used for untargeted metabolomic analysis. Data from both positive and negative ion modes were collected to improve the metabolite coverage. A total of 3396 ions (RSD ≤ 30%) in the ESI+ mode (collecting positively charged ions) and 955 ions (RSD ≤ 30%) in the ESI− mode (collecting negatively charged ions) were detected. The base peak ion (BPC) chromatogram in Figure S2 shows that the selected sample from each group has a narrower peak and a large peak capacity. Principal component analysis score plots of the QC sample and all experimental samples were used to observe the overall distribution of samples between the exercise and control groups, as well as the stability of the entire analytical process. The tightly clustered QC samples indicate the stability of detection and the high reproducibility of the acquired data.

PLS-DA was used as a supervised approach to visualize the clustering and separation of metabolites between the two groups. As shown in Figure S3, there was a clear separation between the exercise group and the control group in both the positive (Figure S3A) and negative (Figure S3B) ionization modes. This indicates that significant differences existed between the two groups. To evaluate the quality of the PLS-DA model, 200 response permutation tests were performed, and the model parameters are shown in Table S3.

As shown in Table S3, the values of R2Y(cum) and Q2(cum) were 0.99 and 0.52 in the ESI+ mode and 0.98 and 0.30 in the ESI− mode, respectively. These values indicate that the fitness and prediction of the PLS-DA model were good. The Q2 intercept values determined by permutation testing were 0.00 to −0.89 for the ESI+ mode (Figure S4A) and 0.00 to 0.97 for the ESI− mode (Figure S4B), which proves that the model presented low overfitting, as the Q2 regression line in red had a negative intercept.

### 3.5. DMs between the Two Groups

**Figure 4:**
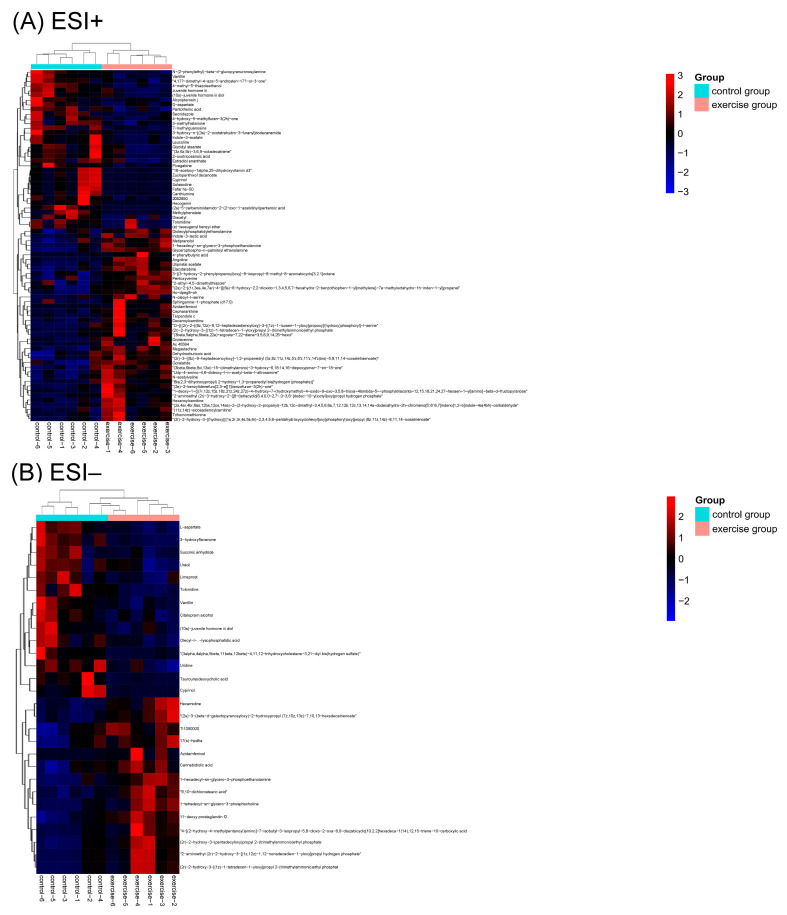
Heat map of the differential metabolites (DMs) in the positive electron spray ionization (ESI+) and negative electron spray ionization (ESI−) modes (**A**,**B**). Red color represents relative increase in abundance, and blue color represents relative decreases in abundance. Color codes are indicated in the color bar on the right side.

A total of 75 DMs in the ESI+ mode and 28 DMs in the ESI− mode (Table S4) were determined with VIP values ≥ 1, FC ≥ 1.20 or ≤0.83, and *p*-values < 0.05. In the ESI+ mode, 41 metabolites were upregulated, i.e., *N*-acetyl valine, and 34 were downregulated, i.e., D-aspartate and pantothenic acid, in the exercise group. In the ESI− mode, 14 metabolites were upregulated, i.e., 9,10-dichlorostearic acid, and 14 were downregulated, i.e., uracil and uridine, in the exercise group. Detailed information on DMs is presented in Table S4. A heat map (Figure 4) was constructed to visualize the changes in the abundance of DMs between the exercise and control groups.

### 3.6. Pathway Analysis

**Figure 5:**
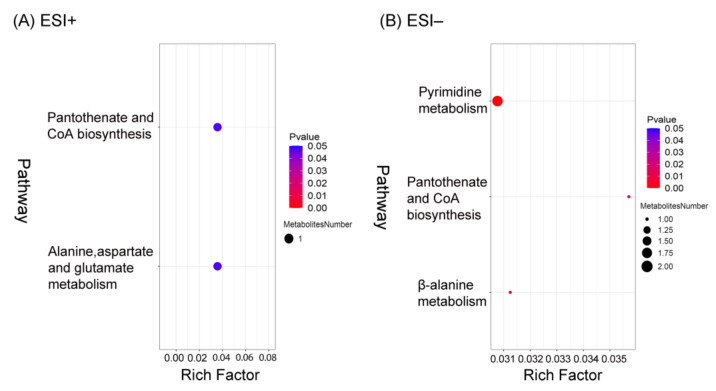
Bubble plots for metabolic pathway enrichment analysis of the differential metabolites (DMs) in the positive electron spray ionization (ESI+) and negative electron spray ionization (ESI−) modes (**A**,**B**). The *y*-axis is the enriched metabolic pathway, and the *x*-axis is the enrichment factor (RichFactor). The larger the value of RichFactor, the greater the proportion of DMs annotated to the pathway. The size of dots represents the number of DMs annotated to a given pathway.

To explore the potential metabolic pathways affected by exercise, 75 DMs in the ESI+ mode and 28 DMs in the ESI− mode were subjected to pathway enrichment analysis using the KEGG database. Ten pathways were enriched, of which four were enriched significantly with *p*-values < 0.05 (Figure 5). These four pathways comprised alanine, aspartate, and glutamate metabolism; pantothenate and coenzyme A (CoA) biosynthesis; pyrimidine metabolism; and β-alanine metabolism (Table S5). As shown in Table 1, there were four DMs enriched in the metabolic pathways identified in this study.

### 3.7. Analysis of Transcriptomic Data

In total, 44.75 and 44.68 million high-quality 150 bp clean reads were mapped to the D. rerio genome from the exercise and control groups, respectively. Quality analysis of the transcriptomic data showed that the exercise and control groups had an average of 96.61% and 96.82% clean reads, Q20 of 97.57% and 97.92%, and Q30 of 93.38% and 94.15%, respectively. The average mapping rates of genes were 87.12% and 87.18% in the exercise and control groups, respectively (Table S6). This indicates that the sequencing quality was good and that the subsequent transcriptomic analysis results were reliable.

**Figure 6:**
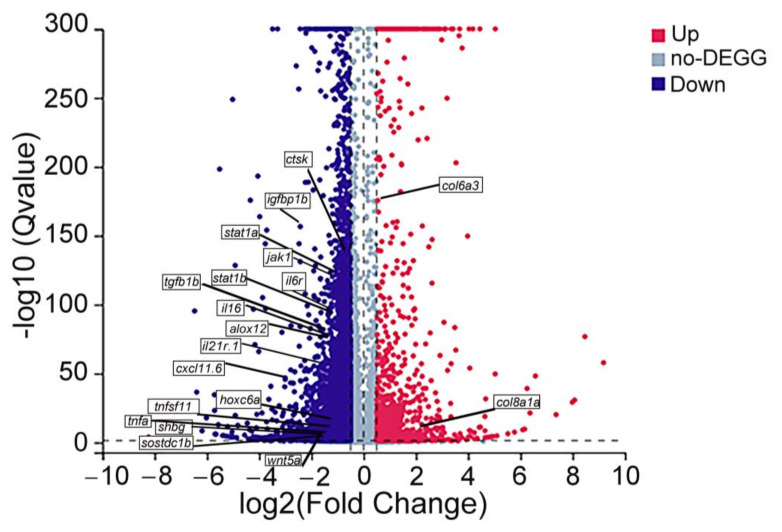
Volcano map of differentially expressed genes (DEGs). The dots represent downregulated (blue) and upregulated (red) DEGs. Genes without difference between the two groups are labeled in purple–gray. The genes in the boxes are reported genes related to bone metabolism.

A total of 9335 DEGs were identified between the exercise and control groups (Figure 6). The statistical significance was defined as *q* < 0.05 (BH) and |Log2FC| > 0.5. Detailed information on the identified 9335 DEGs is shown in Table S7. Compared with the control group, 1088 genes were significantly upregulated and 8247 genes were significantly downregulated in the exercise group. Some previously reported bone-related genes were also observed the significantly differential expressions between the two groups. *Col6a3* (Log2FC = 0.54, *q* = 2.99 × 10^−176^) and *col8a1a* (Log2FC = 2.09, *q* = 9.43 × 10^−5^) were upregulated, and *igfbp1b* (Log2FC = −2.42, *q* = 2.64 × 10^−157^), *ctsk* (Log2FC = −0.65, *q* = 2.55 × 10^−134^), *il6r* (Log2FC = −0.82, *q* = 6.75 × 10^−98^), *tnfsf11* (Log2FC = −1.14, *q* = 4.02 × 10^−7^), *sostdc1b* (Log2FC = −1.77, *q* = 6.18 × 10^−4^), etc., were downregulated in the exercise group. Detailed information on these reported bone-related DEGs is shown in Table S8.

Among the 9335 DEGs, 35 genes (after removing duplicates) were coenriched with the 103 abovementioned DMs in the four metabolic pathways (Table 2): 12 genes in the alanine, aspartate, and glutamate metabolic pathway; 2 genes in the pantothenate and CoA biosynthesis pathway; 17 genes in the pyrimidine metabolic pathway; and 5 genes in the β-alanine metabolic pathway.

### 3.8. Integrated Analysis of Metabolomic and Transcriptomic Data

**Figure 7:**
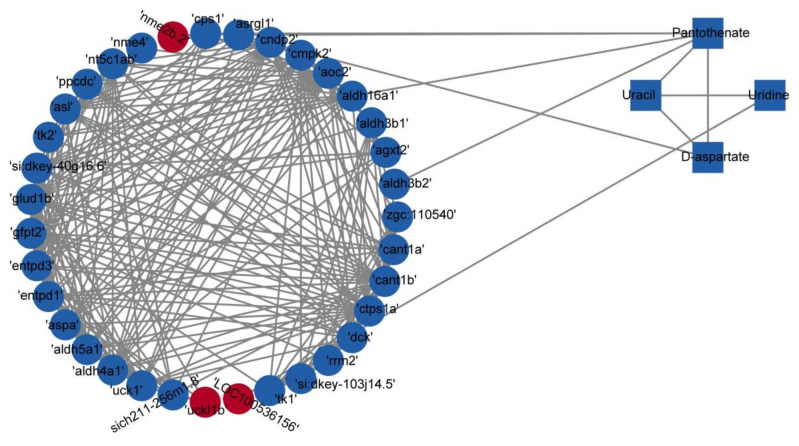
Correlation-based networks of metabolites and genes in the four identified pathways. Rectangles represent metabolites, and circles represent genes. Red indicates upregulation, and blue indicates downregulation.

**Figure 8:**
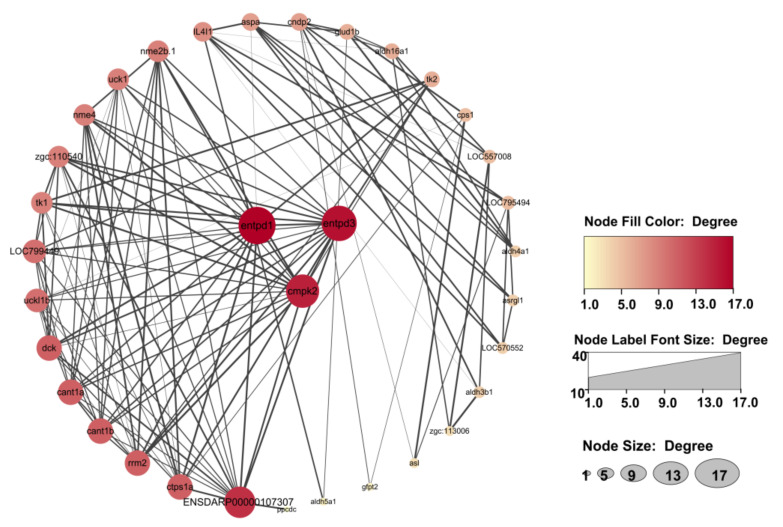
Protein–protein interaction network analysis of 35 differentially expressed genes in the four identified pathways. *Entpd3*, *entpd1*, and *cmpk2* located in the center of the network are core genes.

**Figure 9:**
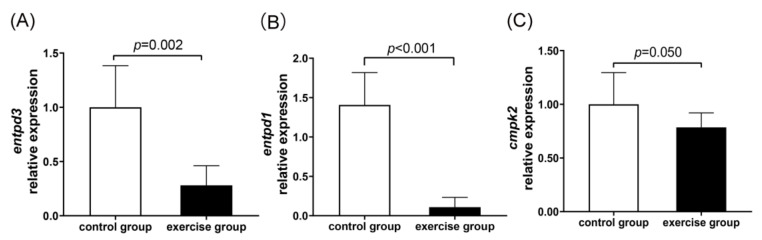
The results of mRNA expression by qRT-PCR for *entpd3* (**A**), *entpd1* (**B**), and *cmpk2* (**C**, the core genes) in the control and exercise groups. All values are presented as mean and standard deviation. Student’s *t* test was used to compare the differences between the two groups.

**Figure 10:**
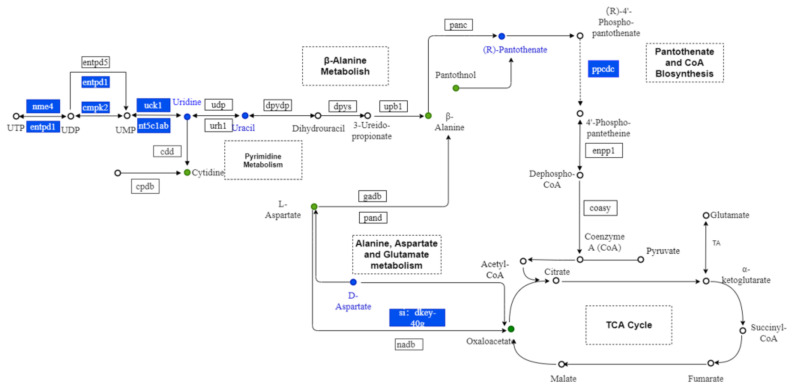
Metabolic maps of alanine, aspartate, and glutamate metabolism, pantothenate and CoA biosynthesis; pyrimidine metabolism; and β-alanine metabolism. Dots represent the metabolites. Boxes represent the genes. Blue indicates downregulation.

An integrated analysis of metabolomic and transcriptomic data was performed using the KEGG database. Correlation-based networks of metabolites and genes in the four pathways were built using Cytoscape 3.9.1. (Figure 7). Close correlation (r > 0.50, *p* < 0.05) of metabolites and genes suggested that the selected metabolites, genes, and pathways in this study were credible. To screen the key genes among the 35 genes across the four metabolic pathways, a protein–protein interaction network analysis of the 35 genes was performed using the STRING database and Cytoscape 3.9.1 (Figure 8). The results show that the genes *entpd3*, *entpd1*, and *cmpk2* were in the core position of the interaction network. qRT-PCR was further conducted to verify the differential expression of mRNAs of *entpd3*, *entpd1*, and *cmpk2* between the exercise and control groups. As illustrated in Figure 9, the qRT-PCR results for the three genes were similar to the results of RNA-seq. For all three genes, e.g., *entpd3*, *entpd1*, and *cmpk2*, the mRNA expression levels were lower in the exercise group (*p* ≤ 0.05). Figure S5 shows the crossover between the metabolomic and transcriptomic analyses. Additionally, to show the relationship between the genes and metabolites from the four pathways more clearly, a comprehensive gene–metabolism network was constructed (Figure 10).

## 4. Discussion

In this study, we used metabolomic and transcriptomic analyses to explore the changes in metabolic pathways, metabolites, and expression of core genes related to improved bone phenotype in zebrafish subjected to an exercise intervention. A total of 103 DMs were found, of which 55 were upregulated and 48 were downregulated in the bone tissues of zebrafish subjected to counter-current swimming training. These DMs were enriched in 10 metabolic pathways, among which alanine, aspartate, and glutamate metabolism; β-alanine metabolism; pyrimidine metabolism; and pantothenate and CoA biosynthesis were significantly enriched. In addition, 35 genes were found to be enriched in these four metabolic pathways, three of which, namely *entpd3*, *entpd1*, and *cmpk2*, which are enriched in pyrimidine metabolism, could be the core genes involved in the regulation of exercise-induced improvements in bone parameters.

Exercise can regulate bone metabolism and has a positive effect on bone mass [41]. Compared with the control group, the zebrafish in the exercise group in this study had higher values of bone mass and mineralized matrix after 8 weeks of counter-current swimming training to simulate resistance movement. These results are similar to those reported in previous studies on zebrafish. Fiaz et al., found that two weeks of swim training accelerated chondrogenesis and osteogenesis of the cranial, axial, and appendicular skeleton in zebrafish [20]. Suniaga et al., found that four weeks of swim exercise significantly increased the bone volume, the number of osteoblasts per bone perimeter, and the bone mass in zebrafish [21]. A study in rats also revealed that ladder-climbing resistance training significantly increased the bone mass of the left tibia and femur [42]. In 2020, a systematic review and meta-analysis revealed that dynamic resistance exercise can maintain bone mass in postmenopausal women [43]. Furthermore, the results from our biochemical analysis and histological staining suggest that the increased bone mass in the zebrafish of the exercise group may be due to enhanced osteoblast activity and inhibited osteoclast activity, which can facilitate bone tissue mineralization and promote bone formation. These findings are supported by previous studies. A study of goldfish found that after three days of swimming exercise, the Alp activity in scales increased significantly [44]. In salmon, Totland et al., found that sustained swimming significantly upregulated the mRNA expression level of the alp gene [45]. Studies in rats also reported that running exercise promoted bone mass gain and prevented bone loss by increasing ALP activity and decreasing TRAP activity [46,47]. Studies have shown that exercise can improve bone formation by regulating multiple pathways to promote osteoblast differentiation and inhibit osteoclast activity [48,49]. In this study, some genes in several well-known pathways related to bone and mineral metabolism, i.e., the Wnt signaling pathway, were also observed to have different expressions between the two studied zebrafish groups.

In our study, compared with the control group, the exercise group showed considerably decreased D-aspartate levels in the alanine, aspartate, and glutamate metabolic pathway, together with increased bone mass. This metabolic pathway has been found to be related to bone mass, and the relationship between glutamate and bone mass has received considerable research attention [50]. In 601 healthy Taiwanese women, plasma glutamate levels were shown to be significantly associated with low BMD [51]. Glutamate receptors are widely expressed in osteoblasts, osteoclasts, and osteocytes. Glutamate may promote bone resorption through the expression of the major type of glutamate receptor, namely the *N*-methyl-D-aspartame-type glutamate receptor, in osteoclasts [52]. Aspartate and glutamate are both neurotransmitters that bind to this receptor [53]. D-aspartate may have similar effects on osteoclasts as glutamate. In addition, glutamate is used as an energy source through its conversion to α-ketoglutarate in the tricarboxylic acid cycle [54]. Osteoblasts have highly active metabolism and high energy expenditure due to exercise-induced osteoblast differentiation. The glutamate concentration in osteoblasts is low, probably due to the interconversion between glutamate and α-ketoglutarate [53]. D-aspartate is involved in glutamate metabolism. The decreased D-aspartate concentration in osteoblasts may also be explained by a faster metabolism in osteoblasts attributable to exercise-induced bone formation [53]. The molecular mechanism underlying the role of D-aspartate in bone metabolism remains unclear and warrants further studies.

The bone tissue of zebrafish in the exercise group showed decreased levels of uracil, which is involved in two pathways, namely β-alanine metabolism and pyrimidine metabolism. In 2021, Rui et al., found that serum uracil levels were inversely associated with BMD in 517 peri- and postmenopausal Chinese women [55]. Uracil can be converted to dihydrouracil by dihydropyrimidine dehydrogenase, which can be further converted to β-alanine. A study of 148 postmenopausal women found that β-alanine is a potential biomarker of osteoporosis and that β-alanine levels in urine samples from the osteoporosis group were upregulated compared with the normal bone mass group [56]. Upregulation of the β-alanine metabolic pathway was also observed in ovariectomized rats compared with sham rats, a commonly used animal model for osteoporosis [57]. All of these findings indicate that the β-alanine metabolic pathway may be involved in the regulation of exercise-induced improvements in bone mass.

Both uridine and uracil are involved in the pyrimidine metabolic pathway and can be interconverted by 5-nucleotidase. A study found that uridine levels in rat liver tissue were significantly decreased in the exhaustive exercise group compared with the control group [58]. Their results support our finding that uridine was significantly downregulated in the exercise group compared with the control group. In 2022, Wang et al., found that compared with the sham group, uridine in bone tissue was significantly upregulated in ovariectomy-induced osteoporosis mice [59]. Increased uracil and uridine levels were also observed in mature osteoblasts [60], suggesting that both of these pyrimidines are involved in the differentiation and maturation of osteoblasts. In addition, *entpd3*, *entpd1*, and *cmpk2* were found to be the core genes in this study, all of which were enriched in pyrimidine metabolism. Consistent with the metabolomic data, all of these genes were downregulated in the exercise group compared with the control group. This indicates that *entpd3*, *entpd1*, and *cmpk2* may be important genes involved in mediating exercise-induced improvements in bone mass.

Compared with the control group, the exercise group showed reduced pantothenate levels. Pantothenate was identified to be involved in the biosynthesis of pantothenic acid and CoA. β-Alanine from uracil can also be converted to pantothenate by pantothenate synthetase. Consistent with our finding, a study on endurance athletes reported that pantothenic acid levels in athletes’ blood were evidently lower than the reference ranges [61]. The impacts of pantothenic acid on bone health remain uncertain due to limited studies. In addition to lower pantothenate levels, the exercise group in the present study had a higher bone mass than that of the control group. This is inconsistent with the results reported in another study. In 2021, Zhang et al., found that plasma pantothenate levels were positively correlated with femoral neck BMD in 1552 participants aged 30 to 82 years [62]. The reason for this inconsistency may be the differences in sample sources. Plasma is a non-organ-specific tissue that reflects complex biochemical processes throughout the body, whereas the bone tissue metabolomic data in this study reflect aberrant metabolism occurring specifically in bone [63]. The relationship between pantothenate in bone tissue and bone mass needs to be further investigated.

To the best of our knowledge, this is the first study to explore the exercise-induced metabolic changes in bone. Non-targeted LC-MS metabolomic analysis combined with RNA-seq transcriptomic analysis revealed extensive metabolic changes in the bone tissue of zebrafish after 8 weeks of counter-current swimming training. These non-targeted approaches allowed for the identification of complete alterations in metabolites compared with targeted techniques with limited profiling capability. Notably, due to the physical properties of bone tissue, metabolite extraction from it is more difficult than from other tissues such as plasma and urine, leading to a small number of DMs being identified in our study. However, plasma or urine metabolic profiling reflects the metabolite changes in the whole body, whereas the bone tissue metabolic profiling conducted in our study reflects the metabolic changes occurring specifically in bone tissue. In addition, a protein–protein interaction network and the gene–metabolism network were constructed based on the prediction analysis, requiring further experimental validation in the future.

## 5. Conclusions

This study demonstrates that exercise training may promote bone formation and enhance bone mass. Four metabolic pathways—alanine, aspartate, and glutamate metabolism; β-alanine metabolism; pyrimidine metabolism; and pantothenate and CoA biosynthesis—were identified to be downregulated in the bone tissue of zebrafish subjected to 8-week swimming training. Of the genes enriched in these four metabolic pathways, *entpd3*, *entpd1*, and *cmpk2* were enriched in pyrimidine metabolism and may be the core genes involved in promoting exercise-induced improvements in bone mass. Our assessment of changes in metabolites illustrates the complexity of exercise-induced improvements in bone mass and bone health and may provide valuable information for future studies.

## Figures and Tables

**Table 1:** Differential metabolites enriched in the four identified pathways.

Pathway	Name	KEGG ID	Mode	Expression Level		VIP	FC	*p*. Value	Regulation
				Control Group	Exercise Group				
Alanine, aspartate, and glutamate metabolism	D-aspartate	C00402	ESI+	5.34 × 10^7^ ± 2.52 × 10^7^	3.04 × 10^7^ ± 1.67 × 10^7^	1.46	0.57	0.045	down
Pantothenate and CoA biosynthesis	Pantothenate	C00864	ESI+	1.27 × 10^8^ ± 2.27 × 10^7^	8.96 × 10^7^ ± 2.33 × 10^7^	1.13	0.71	0.022	down
Pantothenate and CoA biosynthesis; pyrimidine metabolism; β-alanine metabolism	Uracil	C00106	ESI−	4.44 × 10^7^ ± 9.00 × 10^6^	3.32 × 10^7^ ± 6.69 × 10^6^	1.00	0.75	0.048	down
Pyrimidine metabolism	Uridine	C00299	ESI−	2.59 × 10^7^ ± 4.91 × 10^6^	1.82 × 10^7^ ± 4.17 × 10^6^	1.13	0.70	0.015	down

Notes: ID, identification; VIP, variable importance in projection; FC, fold change.

**Table 2:** The 35 genes coenriched with differential metabolites in identified metabolic pathways.

Name	Gene ID	Average FPKM		Log2FC	*q*. Value	Regulation
		Control Group	Exercise Group			
Alanine, aspartate, and glutamate metabolism						
*si:dkey-40g16.6*	337166	2.34 ± 1.66	1.13 ± 1.56	−1.07	1.51 × 10^−15^	down
*glud1b*	373092	159.80 ± 56.49	112.42 ± 29.39	−0.54	<1.00 × 10^−300^	down
*asl*	393423	12.97 ± 3.68	7.90 ± 3.175	−0.76	8.17 × 10^−37^	down
*aldh4a1*	394133	23.90 ± 17.11	15.72 ± 12.01	−0.64	5.39 × 10^−57^	down
*asrgl1*	541402	9.65 ± 1.95	6.57 ± 3.10	−0.58	2.19 × 10^−15^	down
*cps1*	555623	0.53 ± 0.38	0.32 ± 0.19	−0.66	4.65 × 10^− 5^	down
*aspa*	557232	4.67 ± 6.14	2.63 ± 3.30	−0.87	2.21 × 10^−12^	down
*aldh5a1*	565235	6.80 ± 3.46	4.68 ± 3.26	−0.56	8.02 × 10^−17^	down
*gfpt2*	569945	1.84 ± 1.66	1.15 ± 1.28	−0.69	9.76 × 10^−7^	down
*si:dkey-103j14.5*	570552	0.46 ± 0.62	0.26 ± 0.26	−0.90	5.62 × 10^−6^	down
*agxt2*	619269	36.68 ± 22.26	25.92 ± 29.65	−0.51	2.48 × 10^−56^	down
*si:ch211-56m1.8*	795494	1.25 ± 0.72	0.86 ± 0.52	−0.58	1.68 × 10^−8^	down
Pantothenate and CoA biosynthesis						
*LOC100536156*	100536156	0.61 ± 0.77	1.53 ± 2.23	1.26	6.81 × 10^−8^	up
*ppcdc*	393753	7.05 ± 1.83	4.76 ± 1.10	−0.61	4.21 × 10^−13^	down
Pyrimidine metabolism						
*LOC100536156*	100536156	0.61 ± 0.77	1.53 ± 2.23	1.26	6.81 × 10^−8^	up
*nme2b.2*	30084	17,455.26 ± 18	32,248.48 ± 21	0.98	<1.00 × 10^−300^	up
*uckl1b*	558466	11.70 ± 3.13	21.01 ± 14.84	0.80	4.08 × 10^−100^	up
*entpd3*	100005551	40.96 ± 29.61	13.65 ± 9.90	−1.63	<1.00 × 10^−300^	down
*rrm2*	30733	9.68 ± 10.39	4.45 ± 4.27	−1.13	5.09 × 10^−65^	down
*ctps1a*	322089	10.92 ± 6.16	7.30 ± 2.21	−0.61	1.00 × 10^−27^	down
*tk1*	327590	0.95 ± 0.81	0.48 ± 0.36	−1.00	3.54 × 10^−8^	down
*nme4*	394170	1.17 ± 0.88	0.67 ± 0.31	−0.82	1.93 × 10^−3^	down
*cant1a*	406558	12.62 ± 6.37	8.66 ± 3.85	−0.57	7.55 × 10^−24^	down
*tk2*	437016	6.14 ± 1.63	4.35 ± 1.54	−0.51	4.89 × 10^−8^	down
*entpd1*	445151	19.69 ± 7.60	13.17 ± 5.73	−0.61	3.22 × 10^−54^	down
*cant1b*	445201	16.76 ± 7.31	8.55 ± 4.27	−1.00	5.64 × 10^−87^	down
*uck1*	447928	4.76 ± 2.91	3.15 ± 2.51	−0.62	4.26 × 10^−6^	down
*dck*	474325	3.45 ± 1.97	1.85 ± 0.98	−0.89	1.20 × 10^−7^	down
*zgc:110540*	541538	2.72 ± 2.44	1.74 ± 0.71	−0.67	2.08 × 10^−6^	down
*cmpk2*	570478	2.00 ± 1.34	0.86 ± 0.26	−1.26	3.90 × 10^−16^	down
*nt5c1ab*	799449	1.46 ± 1.04	0.86 ± 0.84	−0.89	6.11 × 10^−8^	down
β-Alanine metabolism						
*aldh3b1*	282559	3.51 ± 3.03	2.39 ± 1.62	−0.57	9.81 × 10^−8^	down
*cndp2*	327288	21.54 ± 12.27	13.88 ± 6.10	−0.64	3.11 × 10^−56^	down
*aldh16a1*	492710	14.81 ± 5.35	9.87 ± 4.32	−0.62	2.47 × 10^−50^	down
*aoc2*	541429	84.09 ± 53.64	36.07 ± 14.10	−1.28	<1.00 × 10^−300^	down
*aldh3b2*	557008	1.76 ± 0.37	1.18 ± 0.64	−0.61	1.48 × 10^−8^	down

Notes: ID, identification; FPKM, fragments per kilobase of exon model per million mapped fragments; FC, fold change.

## Data Availability

The RNA-seq data reported in this artical is available in Sequence Read Archive (SRA) PRJNA949785 (https://www.ncbi.nlm.nih.gov/bioproject/PRJNA949785). Requests for the other data may be directed to the corresponding author and are subject to institutional data use agreements.

## Supplementary Materials

The following supporting information can be downloaded at: https://www.mdpi.com/article/10.3390/nu15071694/s1, Figure S1: Diagram of the two-channel swim tunnel; Figure S2: Base peak chromatograms in the exercise group and the control group in positive ion mode (ESI+) and negative ion mode (ESI−); Figure S3: Score graph of the partial least squares method discriminant analysis model in positive electron spray ionization (ESI+) and negative electron spray ionization (ESI−) modes; Figure S4: Partial least squares method discriminant analysis model response permutation testing graph in positive electron spray ionization (ESI+) and negative electron spray ionization (ESI−) modes; Figure S5: Crossover between the metabolomic and transcriptomic analyses; Table S1: The distribution for each zebrafish in the metabolomic and transcriptomic analyses; Table S2: Information on the primer sequences of *entpd3*, *entpd1*, *cmpk2*, and *β-actin*; Table S3: Parameters for evaluation of the partial least squares discriminant analysis (PLS-DA) quality of bone samples; Table S4: Detailed information on differential metabolites in the ESI+ mode and ESI− modes; Table S5: Metabolic pathway of differential metabolites in this study; Table S6: Statistics of the transcriptome sequencing data in this study; Table S7: Detailed information on the 9335 differentially expressed genes identified in this study; Table S8: Bone-related differentially expressed genes between the control group and the exercise group.
