# Supplementary material for: Integration of Metabolomics and Transcriptomics to Reveal the Metabolic Characteristics of Exercise-Improved Bone Mass
Source: Nutrients. 2023 Mar 30;15(7):1694. doi: 10.3390/nu15071694 (PMC10097349; doi:10.3390/nu15071694)
Supplement: Supplementary file 1 [file nutrients-15-01694-s001.zip › Supplementary Figures.pdf]

Supplementary Figures

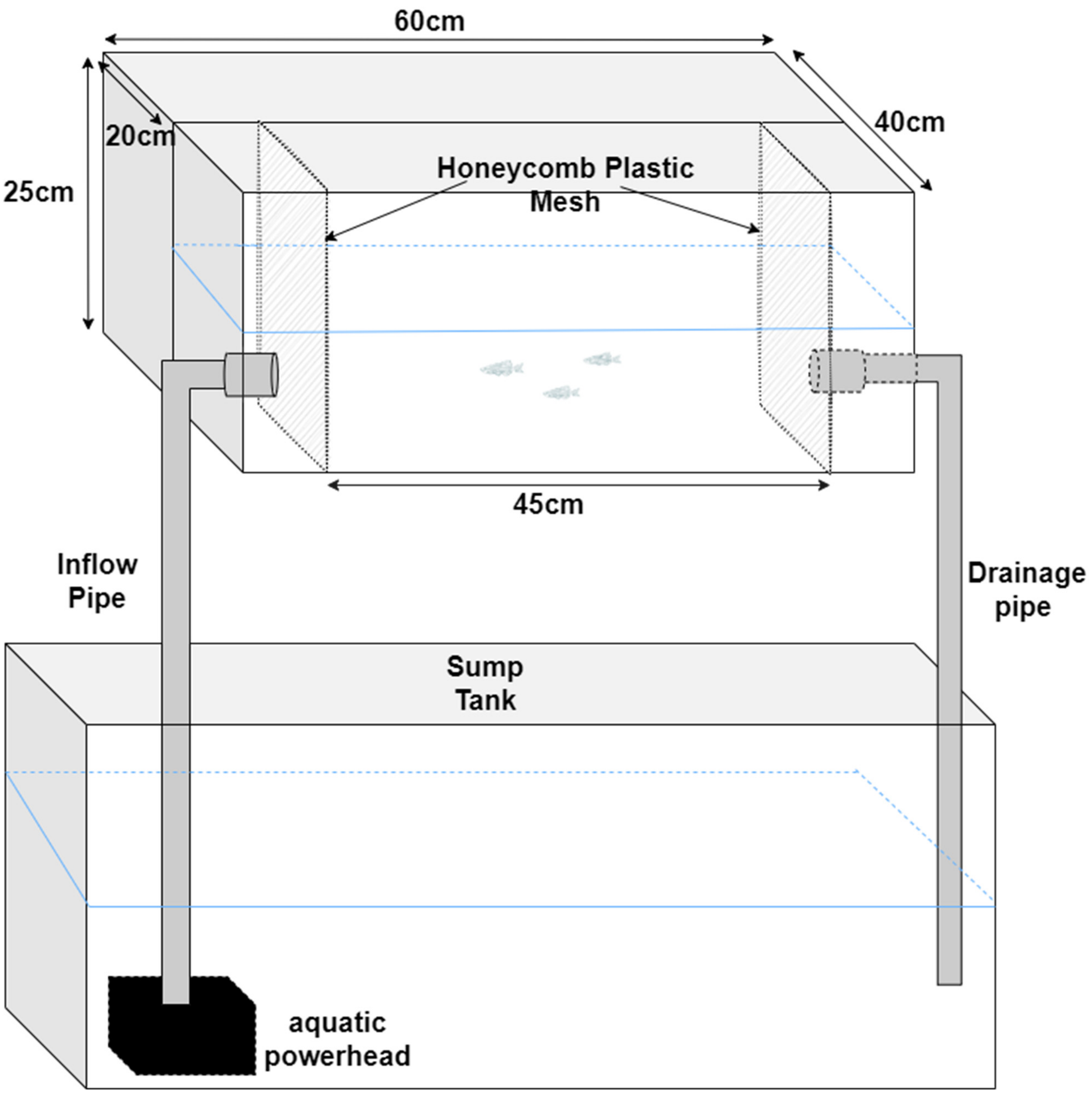

Supplementary Figure S1. Diagram of the two-channel swim tunnel. The swim tunnel is made of the transparent plastic. Each channel has an inflow pipe at one end and a drainage pipe at the other. Both the inlet and the outlet of the swim tunnel are blocked by a honeycomb plastic mesh that allows a smooth flow of water and keeps the fish confined to a 45 cm long area. The water in each channel is

kept at a constant depth of 15 cm. Water is delivered to each channel via an aquatic powerhead in the sump tank located underneath each channel.

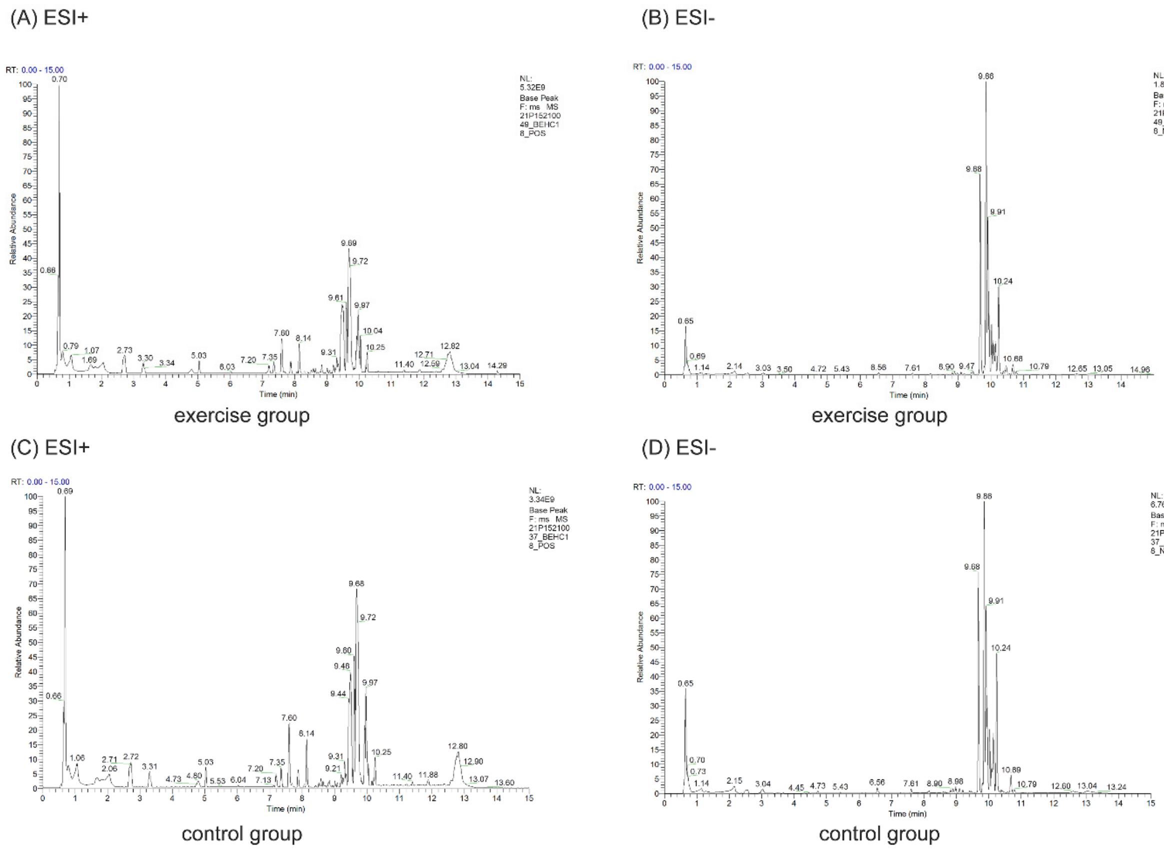

Supplementary Figure S2. Base peak chromatograms in the exercise group and the control group in positive ion mode (ESI+) and negative ion mode (ESI-). **(A)**: exercise group in ESI+; **(B)**: exercise group in ESI-; **(C)**: control group in ESI+; **(D)**: control group in ESI-.

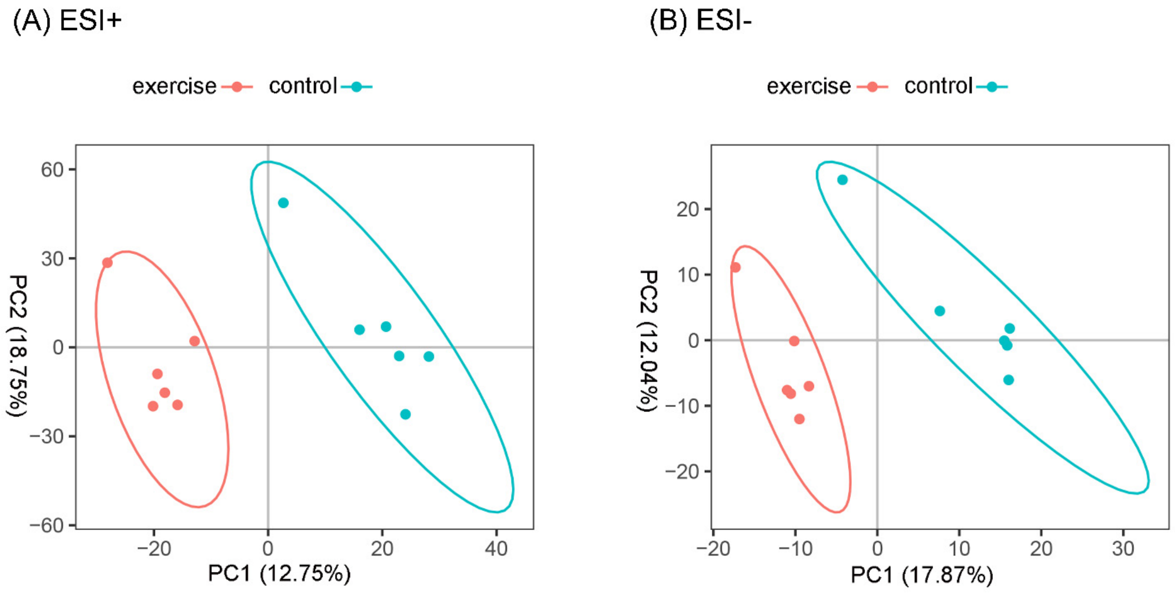

Supplementary Figure S3. Score graph of the Partial Least Squares Method Discriminant Analysis model in positive electron spray ionization (ESI+) and negative electron spray ionization (ESI-) modes. The x-axis is the first principal component. The y-axis is the second principal component. The number in the parentheses is the percentage of the explanation on overall variance of the specific principal component.

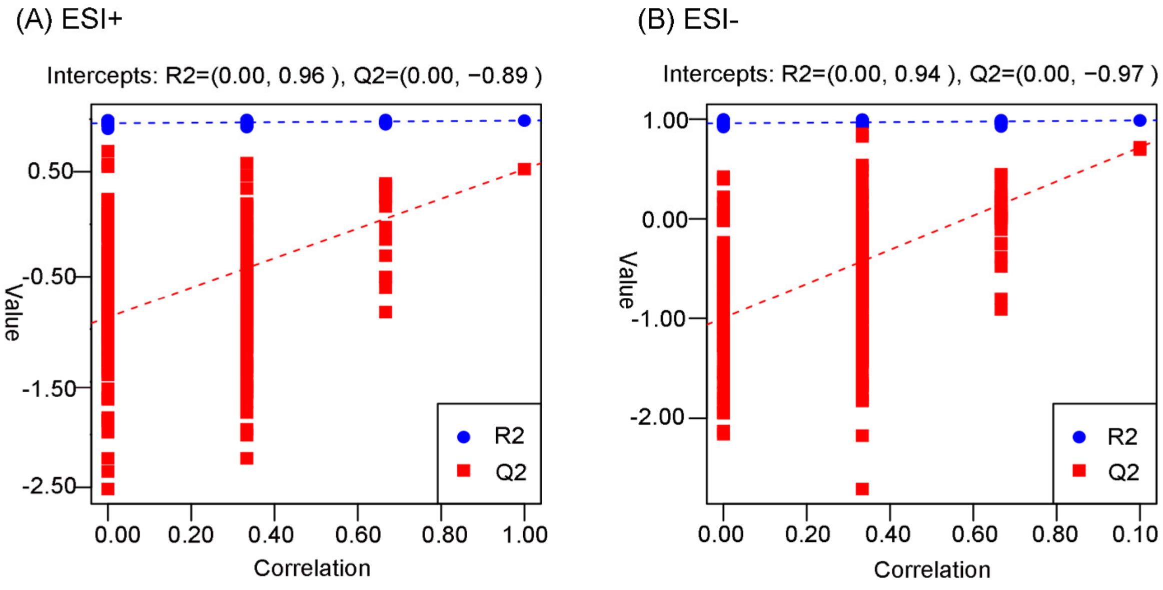

Supplementary Figure S4. Partial Least Squares Method Discriminant Analysis model response permutation testing graph in positive electron spray ionization (ESI+) and negative electron

spray ionization (ESI-) modes. The x-axis is the value of correlation. The y-axis is the value of R2 and Q2.

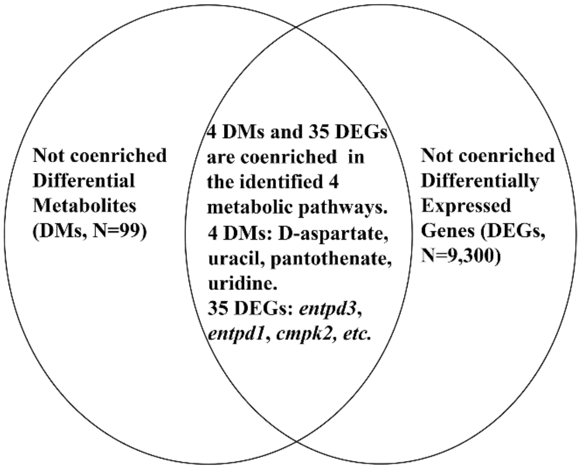

Supplementary Figure S5. Crossover between the metabolomic and transcriptomic analyses. The ellipse on the left represents the 103 differential metabolites (DMs) between the exercise group and the control group. The ellipse on the right represents the 9,335 differentially expressed genes (DEGs) between the two groups. The overlapping part of the two ellipses indicates that there are 4 DMs and 35 DEGs co-enriched in the identified 4 metabolic pathways.
